# Proton Conducting Organic-Inorganic Composite Membranes for All-Vanadium Redox Flow Battery

**DOI:** 10.3390/membranes13060574

**Published:** 2023-06-01

**Authors:** Sooraj Sreenath, Nayanthara P. Sreelatha, Chetan M. Pawar, Vidhiben Dave, Bhavana Bhatt, Nitin G. Borle, Rajaram Krishna Nagarale

**Affiliations:** 1 Electro Membrane Processes Laboratory, Membrane Science and Separation Technology Division, CSIR-Central Salt and Marine Chemicals Research Institute, Bhavnagar 364002, India; sooraj4993@gmail.com (S.S.); nayantharabiju2015@gmail.com (N.P.S.); chetanpawar085@gmail.com (C.M.P.); vidhidave009@gmail.com (V.D.); bhavanabhatt26@gmail.com (B.B.); nitinborle34@gmail.com (N.G.B.); 2 Academy of Scientific and Innovative Research (AcSIR), Ghaziabad 201002, India

**Keywords:** polyvinyl alcohol, silica, sol-gel, metal oxide, tin dioxide, zirconium dioxide, proton-conducting membrane, vanadium redox flow battery

## Abstract

The quest for a cost-effective, chemically-inert, robust and proton conducting membrane for flow batteries is at its paramount. Perfluorinated membranes suffer severe electrolyte diffusion, whereas conductivity and dimensional stability in engineered thermoplastics depend on the degree of functionalization. Herein, we report surface-modified thermally crosslinked polyvinyl alcohol-silica (PVA-SiO_2_) membranes for the vanadium redox flow battery (VRFB). Hygroscopic, proton-storing metal oxides such as SiO_2_, ZrO_2_ and SnO_2_ were coated on the membranes via the acid-catalyzed sol-gel strategy. The membranes of PVA-SiO_2_-Si, PVA-SiO_2_-Zr and PVA-SiO_2_-Sn demonstrated excellent oxidative stability in 2 M H_2_SO_4_ containing 1.5 M VO_2_^+^ ions. The metal oxide layer had good influence on conductivity and zeta potential values. The observed trend for conductivity and zeta potential values was PVA-SiO_2_-Sn > PVA-SiO_2_-Si > PVA-SiO_2_-Zr. In VRFB, the membranes showcased higher Coulombic efficiency than Nafion-117 and stable energy efficiencies over 200 cycles at the 100 mA cm^−2^ current density. The order of average capacity decay per cycle was PVA-SiO_2_-Zr < PVA-SiO_2_-Sn < PVA-SiO_2_-Si < Nafion-117. PVA-SiO_2_-Sn had the highest power density of 260 mW cm^−2^, while the self-discharge for PVA-SiO_2_-Zr was ~3 times higher than Nafion-117. VRFB performance reflects the potential of the facile surface modification technique to design advanced membranes for energy device applications.

## 1. Introduction

Ion-exchange membranes (IEMs) have been known over a century for the separation/purification and energy device applications [1,2,3]. The inherent property requirement for the membrane is the ion selectivity [4,5]. In the case of cation-exchange membranes (CEMs), cations are transferred, whereas in the case of anion-exchange membranes (AEMs), anions are transferred [6]. Fascinatingly, in the last decade, with the explosion of the research into energy storage systems such as redox flow batteries (RFBs) and fuel cells, the selective transfer of protons is the primitive requirement from the membrane [7,8,9,10]. A plethora of research is devoted to the preparation of proton-selective membranes [11,12,13,14,15]. The basic concept involves the introduction of ionic groups such as sulfonic, phosphonic, carboxylic acid, etc., to the polymer backbone [16,17,18]. The controlled and precise degree of anionic functionalization is important to achieve the desired properties. Proton transfer in anion exchange membranes involves the transfer of protons by simple seeping through the membrane [19]. Nafion and other perfluorinated sulfonic acid membranes by Dupont are highly rated due to elevated proton conductivity, as well as robust dimensional and chemical stability [20,21,22]. However, the high cost, electrolyte crossover and low proton conduction at elevated temperatures are its unmet apprehensions [23]. The sulfonation of engineered thermoplastic polymers has attracted much deserving attention due to good proton conduction over a wide temperature range and low manufacturing cost [24]. However, this requires sulfonic acid functionalization, causing improper regioselectivity and degree of sulfonation. The harsh functionalization conditions due to the use of chlorosulfonic acid results in the partial cleavage of the polymer backbone and causes undesirable crosslinking and branching by rearranging the intermediate sulfonic acid functional units into an irreversible branched/crosslinked sulfone [25]. As another disadvantage, there are reports where sulfonic group undergoes ipso-substitution with protons to lower the hydrolytic stability of the membrane [1]. To address these shortcomings, the polymerization of sulfonated aromatic monomers is reported [26], but the use of expensive metal catalyst restricts its large-scale production. Meanwhile, commercial anion exchange membrane preparation methodology involves chloromethylation, employing highly carcinogenic chloro-methyl methyl ether, followed by the amination of engineering thermoplastics [27].

To exclude the sulfonation/amination functionalization strategy, an alternative approach is to infiltrate a polymer matrix with inorganic materials to obtain inorganic–organic composite ion exchange membranes that will have the properties and characteristic features of inorganic and organic compounds [28]. The inherent hydrophilicity, copious reactive hydroxyl groups and low cost of polyvinyl alcohol (PVA) are reasons to select it as a typical host matrix [29]. Among numerous inorganic materials, silica is the most frequently deployed inorganic filler used in the PVA matrix to improve the mechanical strength, thermal stability, water retention capacity and proton conductivity of the membrane [30,31,32]. Incorporating silica into PVA membrane via an in situ solution-gelation (sol-gel) process is a novel approach compared to the direct physical blending [33]. It prevails well-defined interconnecting conducting channels due to the uniform distribution of silica particles [34]. The compatibility of organic and inorganic phases is defined by multifold covalent bonds, hydrogen bonding and electrostatic interactions [35]. The enhancement of proton conductivity in PVA-SiO_2_ composite membranes is induced by different strategies, of which the most common practices involve (a) the incorporation of functionalized silica, (b) the functionalization of host PVA and (c) the blending of PVA-SiO_2_ with charged polymers/nanomaterials [36,37,38]. There are reports in which a combination of the above-mentioned strategies was used to fabricate a high-proton-conducting PVA-SiO_2_ composite separator for energy devices [39,40]. Recently, Hedge et al. developed hybrid membranes by dispersing sulfonated nanosilica into the sulfonated tetraethyl orthosilicate (TEOS)-crosslinked poly(vinyl alcohol) (PVA) matrix for a proton exchange membrane fuel cell (PEMFC) [41]. The PVA and chitosan membrane composite with poly(2-acrylamido-2-methyl-1-propanesulfonic acid) functionalized silica demonstrated good thermal and oxidative stabilities [42]. Beydaghi and co-workers reported that composite proton exchange membranes from PVA and various amounts of phenyl sulfonic acid functionalized nanoporous silica [43]. Murmu et al. fabricated a cost-effective sulfonated poly ether ether ketone (SPEEK)-PVA-Silica organic–inorganic hybrid composite membrane for fuel cell operation [44]. It is worth mentioning that in most of the literature, the presence of an anionic/cationic functional group and a crosslinker has a significant effect on its proton conductivity and the overall application performance of the membranes [45,46,47]. The control over degree of functionalization and crosslinking is critical as the presence of excess functional groups can undermine its mechanical stability, while poor crosslinking affects its membrane forming property.

The aim of this study is to design and synthesize high performance separators, devoid of anionic/cationic functional groups and crosslinkers for VRFB. Herein, we report the surface modification of a PVA-SiO_2_ composite membrane by the acid-catalyzed sol-gel technique. Three membranes were prepared using silica (SiO_2_), zirconium dioxide (ZrO_2_) and tin dioxide (SnO_2_) surface modifications on a PVA-SiO_2_ composite membrane for an all-vanadium redox flow battery (VRFB). The skin layer of the hygroscopic proton-conducting metal oxides on the membrane acted as a barrier for vanadium ions, whereas the nanoscopic interaction of the skin layer with the bulk silica and the PVA matrix created a well-defined conducting channel for the conduction of charge-balancing hydrated protons (hydronium ions) during the vanadium flow battery operation.

## 2. Materials and Methods

### 2.1. Materials

Polyvinyl alcohol (PVA) (molecular weight 85,000 to 124,000, degree of hydrolysis 86.0–89.0%) was purchased from Chemical Drug House (CDH) Pvt. Ltd., Delhi, India. Tetraethyl orthosilicate (TEOS) was received from Research Lab Fine Chem Industries, Maharashtra, India, as a silica precursor. Zirconium oxychloride and tin chloride were obtained from TCI chemicals, India. Liquor ammonia was procured from Rankem chemicals, Haryana, India. Nitric acid (68% purity) and sulfuric acid (assay quality 98.0%) were purchased from Qualigens (Maharashtra, India). De-ionized (DI) water was used for all the experiments.

### 2.2. Methods

#### Preparation of Proton-Conducting PVA-Based Composite Membranes

A transparent solution of PVA (5 g) in DI water (80 mL) was obtained by constant stirring for 3 h at room temperature. Tetraethyl ortho silicate (TEOS) (5 mL) was added slowly to the solution of PVA to obtain an emulsion with constant stirring. After a 15 min time period, ammonia (25 mL) was added to the emulsion solution and stirring was continued for 6 h to obtain a viscous and transparent solution. The resulting thick solution was cast on an acrylic sheet by adjusting the thickness with a doctor blade and allowed to dry under an infrared lamp. The dried membrane, which is labelled as PVA-SiO_2_, was thermally crosslinked at 140 °C for 4 h. The surface modification of the membranes was achieved by dipping in sol-gel mixtures.

The sol-gel mixture was prepared by mixing 1 M nitric acid (20 mL) and TEOS/ZrOCl_2_/SnCl_4_ (2 mL). The membrane was dipped in the mixture at 50 °C for 5 h. After 5 h, the membranes were rinsed with DI water and dried in an air oven at 50 °C for 1 h. Then, the membranes were stored in DI water for further characterizations. The surface-modified PVA-SiO_2_ membranes obtained from the -catalyzed sol-gel method were labelled as PVA-SiO_2_-Si, PVA-SiO_2_-Zr, and PVA-SiO_2_-Sn, respectively.

### 2.3. Characterizations

The surface morphology of the membranes was visualized at the microscopic level using a field emission scanning electron microscope (FE-SEM) on a JSM7100F, Japan, coupled with energy dispersive X-ray (EDX) spectra with an accelerating voltage of 20 kV. The hydroxyl groups present on the membrane were confirmed by Fourier-transform infrared-attenuated total reflectance (FTIR-ATR). The thermal stability of the membranes was analyzed by thermogravimetric analysis, TA instruments 2960 (METTLER TOLEDO, Giessen, Germany), with a temperature range of room temperature to 800 °C under a N_2_ atmosphere. The phase transition of the membrane was detected by differential scanning calorimetry (DSC-Mettler Toledo, Greifensee, Switzerland). The zeta potential values of the membranes were determined using a Zeta Cad streaming current and zeta potential meter, CAD Instruments (Les Essarts-le-Roi, France). The membranes (area 5 × 2.5 cm) were equilibrated with 1 mM potassium chloride (KCl) solution for 24 h. A two membrane sandwich using a Teflon spacer was then fixed in the cell of the instrument to record the steady-state zeta potential values in 1 mM potassium chloride (KCl).

A CHI 700E potentiostat/galvanostat was used to measure the through-plane conductivity and area specific resistance (ASR) of the metal-oxide-coated PVA-SiO_2_ membranes by AC impedance spectroscopy. Briefly, the membranes were conditioned in 1 M sulfuric acid and vanadium electrolyte (1 M V^3+^ and 1 M VO^2+^ in 2 M H_2_SO_4_) for 24 h to obtain acid-doped and vanadium-electrolyte-doped membranes. The excess surface adhered solution on the membrane was soaked with paper cloth before it was stationed between the stainless-steel circular electrodes of an in-house built conductivity measurement cell with an effective area of 1.0 cm^2^. The impedance was measured by applying 0.005 V of amplitude over a frequency range of 1 Hz to 1 MHz in the potentiostat with the help of compatible software uploaded in the system to obtain the resultant impedance spectra. This was further fit in the model to obtain the required resistance of the membranes. Through-plane conductivity and specific area resistance for the membrane were calculated by substituting the effective membrane area ($A_{memembrane}^{effective}$), membrane thickness and corrected specific resistance ($Z_{eff\;\left(corrected\right)}^{High\;Freq.}$) in the formula mentioned below (Equation (1)). The thickness of the membrane can be measured with screw gauge. The blank cell resistance measurements were performed previously such that the results can be normalized to determine the membrane’s true proton conductivity and area specific resistance (ASR). The corrected specific resistance was calculated using the equation below, in which $(Z_{memembrane+cell}^{High\;Frequency}$) and $\left(Z_{blank\;cell}^{High\;Frequency}\right)$ are the overall membrane-cell assembly resistance and blank-cell resistance obtained at high frequency

$$Z_{effective\;\left(corrected\right)}^{High\;Frequency}={\;Z}_{membrane+cell}^{High\;Frequency}-Z_{blank\;cell}^{High\;Frequency}$$

(1) $$\mathrm{Membrane}\;\mathrm{conductivity}\;\left[\sigma \;\left({\mathrm{mS}\;\mathrm{cm}}^{-1}\right)\right]=\frac{\mathrm{Thickness}\;\mathrm{of}\;\mathrm{membrane}\;\left(\mathrm{in}\;\mathrm{cm}\right)}{Z_{effective\;\;\left(corrected\right)}^{High\;Frequency}\;\left(\Omega \right)\times {\;A}_{memnrane}^{effective}\left({\mathrm{in}\;\mathrm{cm}}^{2}\right)}$$

The traditional gravimetric technique was used to determine the sulfuric acid uptake capacity of the membranes. A 2 × 2 cm piece of each membrane was immersed separately in 50 mL 2 M H_2_SO_4_ for 12 h. After gentle wiping of the surface of a membrane with a tissue paper, the membrane could be weighed to obtain the wet weight (*W_wet_*)_._ Then the same pieces of membranes were dried in an air oven at 60 °C and weighed every 1 h until they obtained a constant weight, i.e., dry weight of the membrane (*W_dry_*). The sulfuric acid uptake of the membrane can be calculated using the below-mentioned equation [48].

(2) $$Sulfuric\;acid\;uptake\;\left(\%\right)=\frac{W_{wet}-W_{dry}}{W_{dry}}\times 100$$

The swelling ratio of the synthesized membranes was calculated by immersing a rectangular piece of membrane in 2 M H_2_SO_4_ for 24 h, after which the membrane was gently wiped off to remove excess surface-adhered acid and the length (*L_wet_*) was measured. The dry length (*L_dry_*) of the membrane can be calculated by simply drying the same piece of membrane in an oven at 60 °C. The obtained values were substituted to the following equation.

(3) $$Swelling\;ratio=\frac{L_{wet}-L_{dry}}{L_{dry}}\times 100$$

The oxidative stability of the membrane is an important parameter to explain the capability of the membrane to withstand the extreme oxidative and corrosive environment of a VRFB. It was evaluated by recording its dimensional change and change in mass in every 24 h duration after its immersion in the 2 M H_2_SO_4_ solution containing 1.5 M VO_2_^+^ ions. The study was conducted for 168 h (7 days) [49].

The vanadium ion diffusivity of the synthesized membranes was determined using a two-compartment cell. The permeability of different vanadium species was studied. The membrane was placed between two compartments of the cell, one of which was filled with vanadium salt in H_2_SO_4_ (depletion compartment) and the other compartment with the respective charge neutralizing species in H_2_SO_4_ (enrichment compartment), as reported in the literature [50]. The experiment was carried out with the constant stirring of the two solutions to avoid solute deposition at the membrane surface and, thereby, the reduction in the concentration of solution. A sample was collected from both the compartments at equal time intervals for 24 h, and the vanadium ion concentration was determined using inductively coupled plasma (ICP). The analysis was carried out with an iCAP RQ, ICP-MS instrument (make: thermoscientic), the vanadium ICP standard deputed to obtain standard calibration curve was NIST SRM^®^ 3165, lot 992706. The following equation was used to obtain the permeability (k/L).

(4) $$\ln\left(C_{D0}-C_{E}\right)-\ln\left(C_{D0}\right)=-\frac{2Ak}{VL}t$$

where *C_D_*_0_ is the initial concentration of vanadium ions in the depletion compartment (mol L^−1^), *C_E_* is the concentration of vanadium ions contained in the enrichment compartment (mol L^−1^), *A* is the exposed area of the membrane (cm^2^), *V* is the volume of the compartment (cm^3^), *L* is the thickness of the membrane (cm), *K* is termed as the diffusion coefficient (cm^2^ s^−1^), and *t* is the time duration of the experiment (s).

All of the single cell flow battery studies were carried out on the commercial redox flow battery provided by Research Supporters India (RSI) with an effective area of 25 cm^2^, which consists of two graphite electrodes, brass current collectors, and the outer PVC covering that enables the in and out flow of the electrolyte without coming into contact with the current collector. The synthesized membrane was placed between the graphite electrodes, and in order to ensure a high electrode surface area for the electrochemical redox reactions of the vanadium ions and compactness of the system, carbon felts were incorporated in between the membrane and the electrode. The battery was made leak proof before starting any experiment. The continuous and constant flow of the electrolytes, posolyte is 20 mL 1 M VO^2+^/VO_2_^+^ in 2 M H_2_SO_4_ and anolyte is 20 mL 1 M V^3+^/V^2+^ in 2 M H_2_SO_4_, was ensured with the aid of small dosing peristaltic pumps. The rate capability performance of the membranes was studied at different current densities, i.e., 20, 40, 60, 80 and 100 mA cm^−2^, with a battery tester procured from Neware, India, and the efficiencies of the system were calculated as reported [48,49]. The open circuit voltage was studied with the same battery by charging up the 15 mL 0.25 M vanadium solutions to 1.8 V @ 50 mA cm^−2^ current density, and the system was left as it is for self-discharging up to 1.0 V. The experiment for polarization study was carried out on an indigenously developed redox flow battery with an effective area of 12.5 cm^2^, and the battery was assembled the same as above. To obtain the polarization curve, the battery was completely charged up to 1.8 V to attain 100% state of charge (SOC) and discharged at different current densities, 25 to 450 mA cm^−2^ with voltage noted.

## 3. Results and Discussion

**Figure 1:**
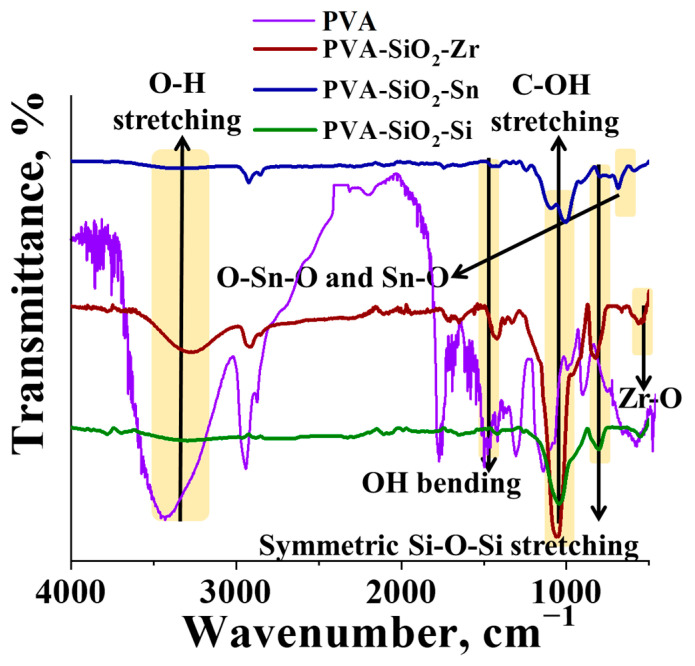
FTIR-ATR spectra of surface-modified PVA-SiO_2_ membranes.

The anionic/cationic functional group free proton-conducting membranes were prepared by surface modification of the PVA-SiO_2_ thermal crosslinked membranes. SiO_2_, ZrO_2_ and SnO_2_ were coated onto the membranes by the acid-catalyzed sol-gel technique. Three self-standing transparent membranes: PVA-SiO_2_-Si, PVA-SiO_2_-Zr and PVA-SiO_2_-Sn, were obtained with an average thickness of ~150 µM. The average thickness of the coated metal oxide was observed to be 1.80–2.00 μM in the cross-sectional SEM images (Figure S1a–c). The thermal crosslinking and metal oxide coating were evident from the FT-ATR spectra of the membranes (Figure 1). The characteristic peaks of PVA and SiO_2_, i.e., ~3400 (O-H stretching), ~1450 (O-H bending) and ~1080 cm^−1^ (C-OH stretching) [51] and symmetric Si-O-Si stretching band at ~815 cm^−1^ and asymmetric Si-O-Si stretching at 1100–1200 cm^−1^, were affirmative for all the membranes [45]. A close introspection of the spectra reveals a minute hump with a certain amount of noise signal instead of a broad peak for O-H stretching, indicating the effective thermal crosslinking of membrane. In addition to these peaks, the characteristic absorption peaks of the Zr-O stretching bond [52] at 440 and 460 cm^−1^ were observed in the spectra of PVA-SiO_2_-Zr, and O-Sn-O and Sn-O stretching bands at 640 and 550 cm^−1^, respectively, were present for PVA-SiO_2_-Sn [53].

**Figure 2:**
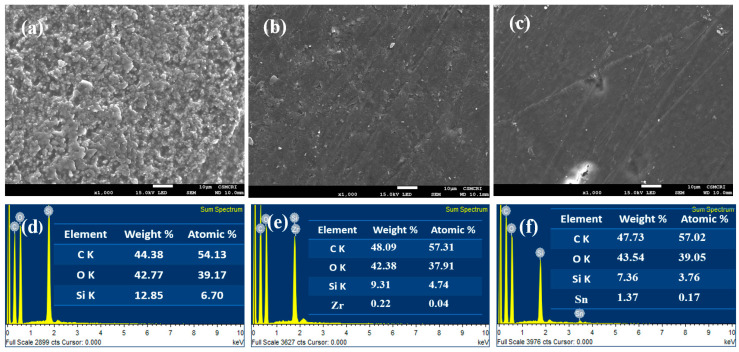
(**a**–**c**) SEM images of the membranes. PVA-SiO_2_-Si, (**b**) PVA-SiO_2_-Zr and (**c**) PVA-SiO_2_-Sn. (**d**–**f**) Energy dispersive X-ray spectra of the membranes (**d**) PVA-SiO_2_-Si, (**e**) PVA-SiO_2_-Zr and (**f**) PVA-SiO_2_-Sn.

The SEM micrographs of PVA-SiO_2_-Si, PVA-SiO_2_-Zr and PVA-SiO_2_-Sn are presented in Figure 2a–c. The membranes were found to be dense and free of surface defects such as pinholes/cracks. For PVA-SiO_2_-Si, the presence of silica particles coated on the surface was clearly visible, the characteristic morphology of silica particles was well interconnected with each other to form a uniform layer of silica on the membrane surface (Figure 2a). In the cases of the PVA-SiO_2_-Zr and PVA-SiO_2_-Sn membranes, no specific morphology was identified for the surface coating. However, the energy dispersive X-ray spectra confirmed the presence of zirconium (Zr) and tin (Sn) on the surface of PVA-SiO_2_-Zr and PVA-SiO_2_-Sn, respectively (Figure 2d–f). Carbon (C), oxygen (O) and silicon (Si) peaks were observed in the EDX spectra of PVA-SiO_2_-Si (Figure 2d), in which the weight percentages of C, O and Si were 44.38, 42.77 and 12.85%, and their corresponding atomic percentages were 54.13, 39.17 and 6.70%. Figure 2e depicts the EDX spectra of PVA-SiO_2_-Zr, the weight percentages of C, O, Si and Zr were 48.09, 42.38, 9.31 and 0.22%, and the recorded corresponding atomic percentages were 57.31, 37.91, 4.74 and 0.04%. The EDX spectra of PVA-SiO_2_-Sn (Figure 2f) confirmed the presence of Sn in addition to C, O and Si. The detected weight percentages of C, O, Si and Sn were 47.73, 43.54, 7.36 and 1.37%, respectively; meanwhile, the corresponding atomic percentages were found to be 57.02, 39.05, 3.76 and 0.17%. Furthermore, the uniform dispersion of SiO_2_, ZrO_2_ and SnO_2_ particles was observed in the elemental mapping of the respective membranes (Supporting Information Figures S2–S4). The SEM surface image with elemental mapping for PVA-SiO_2_-Si reveals the existence of elements C, O and Si with a uniform distribution of Si throughout the membrane matrix (Figure S1). Similarly, the uniform distribution of Zr and Sn was also observed in PVA-SiO_2_-Zr and PVA-SiO_2_-Sn membranes (Figures S2 and S3).

**Figure 3:**
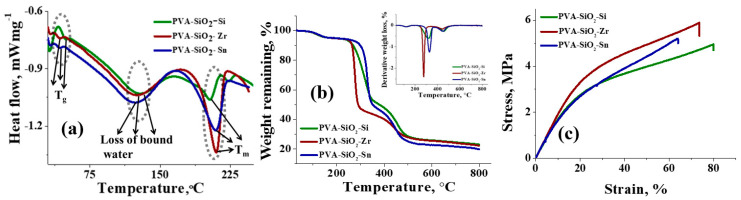
Thermal and mechanical analysis of surface modified PVA-SiO_2_ membranes, (**a**) DSC spectra, (**b**) TGA spectra and (**c**) UTM spectra.

Figure 3 illustrates the DSC response, TGA and mechanical strength analysis of the metal-oxide-coated PVA-SiO_2_ membranes. The DSC spectra of the membranes depicted the glass transition temperature (T_g_) and melting temperature ™, seen in Figure 3a. The obtained values were in accordance with the literature values for PVA-based membranes [9]. The T_g_ values obtained for PVA-SiO_2_-Si, PVA-SiO_2_-Zr and PVA-SiO_2_-Sn were 49.6, 43.2 and 42.4 °C, respectively. The endothermic peak for the membranes in the temperature range 120–130 °C in the DSC spectra corresponds to loss of bound water molecules [36]. Meanwhile, 206, 208 and 209 °C were the T_m_ values for PVA-SiO_2_-Si, PVA-SiO_2_-Zr and PVA-SiO_2_-Sn, respectively. The TGA spectra of the membranes displayed three-step thermal destruction (Figure 3b), which was further confirmed by the first derivative plot of the spectra (Figure 3b inset). The initial weight loss of ~6% was identical for all the membranes in the temperature range 30–200 °C, attributed to the loss of bound water molecules and the thermal solvation of the polymer matrix [54]. The second weight loss, observed just after ~250 °C, corresponds to the dehydration of the metal oxides and thermo-oxidation of the PVA-based polymer matrix [37]. However, the weight loss percentages for the membranes were not the same in this step; it can be seen from the spectra that PVA-SiO_2_-Zr suffered the maximum weight loss of ~48%, whereas there were 46 and 42% weight losses for PVA-SiO_2_-Sn and PVA-SiO_2_-Si. The thermal destruction after 400 °C can be blamed on decomposition of the main chains of the PVA [55]. At this particular step, PVA-SiO_2_-Sn suffered the maximum weight loss of 24%, and for PVA-SiO_2_-Zr and PVA-SiO_2_-Si, the weight losses were 20 and 14%, respectively. The char yield of 23% was obtained for PVA-SiO_2_-Si, meanwhile for PVA-SiO_2_-Zr and PVA-SiO_2_-Sn, it was 22 and 20%, respectively. As a whole, the surface modified PVA-SiO_2_ membranes exhibited acceptable thermal stability for use in different membrane-based applications.

The physicochemical and electrochemical properties of the membranes were evaluated by determination of the sulfuric acid (2 M) uptake, swelling ratio in sulfuric acid (2 M) and through-plane conductivity and area specific resistance (ASR). The sulfuric acid (2 M) uptake of the membranes was estimated gravimetrically. It is necessary for a battery separator to have electrolyte uptake ability, as the soaked-up electrolyte can certainly act as a proton-conducting pathway [56]. As expected, the sulfuric acid (2 M) uptake of the membranes was >85.0% due to the PVA-SiO_2_ based polymer matrix. The highest uptake of sulfuric acid (2 M) was observed for PVA-SiO_2_-Si with a 95.0%, silica coating on the membrane surface, and silica in the bulk polymer matrix enhanced its sulfuric acid uptake [40,45]. Meanwhile, for PVA-SiO_2_-Zr and PVA-SiO_2_-Sn, the uptake was 86.0 and 89.0%, respectively. The inherent solvation ability of metal oxides contributed to the high percentage sulfuric acid uptake of the membranes [57]. It is well-known that high electrolyte uptake in the membrane allows the smooth conduction of ions [58]. However, in a vanadium redox flow battery, a membrane with electrolyte content will raise the alarm for its dimensional stability. For this reason, the membranes were rehydrated to observe the dimensional stability, and we could not observe any deformations as it regained its size and shape. Additionally, the swelling ratios of the membranes in 2 M H_2_SO_4_ were 25, 22 and 20% for PVA-SiO_2_-Si, PVA-SiO_2_-Zr and PVA-SiO_2_-Sn, respectively. Even though the swelling ratio values for the membranes were found to be high, we did not observe any sort of change in dimensions after changing the concentration of the electrolyte solutions, indicating its good utility in the intended application. The dimensional stability of the membranes was further supported by analyzing the mechanical strength in wet conditions (Figure 3c). From the figure, it is observed that the elongation at the break was highest for PVA-SiO_2_-Si with ~80%, while its stress was 4.95 MPa. The elongation at the break for PVA-SiO_2_-Zr, and PVA-SiO_2_-Sn was ~74 and ~64%, and their stress values were 5.90 and 5.20 MPa respectively.

**Figure 4:**
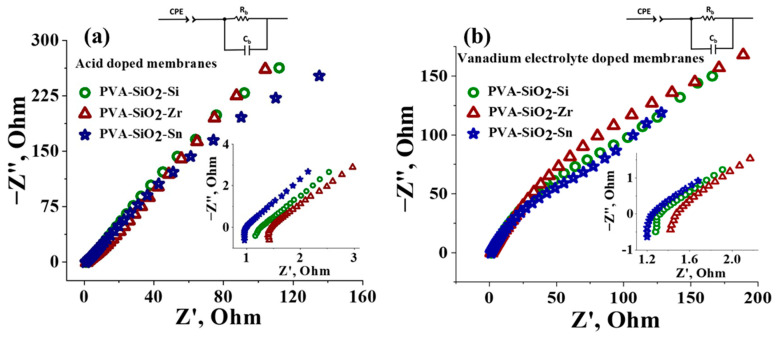
Impedance spectra of surface-modified PVA-SiO_2_ membranes, (**a**) acid-doped membranes and (**b**) vanadium-electrolyte-doped membranes.

The through-plane conductivity and area specific resistance (ASR) of the membranes were calculated from the impedance spectra. Figure 4a,b are the impedance spectra of the acid-doped and vanadium-electrolyte-doped, metal-oxide-coated PVA-SiO_2_ membranes. The calculated conductivity values for the acid-doped PVA-SiO_2_-Si, PVA-SiO_2_-Zr and PVA-SiO_2_-Sn membranes were found to be 15.00, 12.28 and 20.27 mS cm^−1^, respectively, while 1.00, 1.18 and 0.74 ohm cm^2^ was the area specific resistance for acid-doped PVA-SiO_2_-Si, PVA-SiO_2_-Zr and PVA-SiO_2_-Sn membranes, respectively. The bulk of the matrix for all the membranes is PVA-SiO_2_; still, the conductivity and ASR values for the membranes were not identical, suggesting the influence of the metal oxide surface coating on the membranes. Metal oxides were deputed in various studies to facilitate proton transfer pathways [59,60]. The highest conductivity and lowest ASR were recorded for PVA-SiO_2_-Sn, which can be explained based on the excellent proton-conducting feature of SnO_2_ [61]. The inherent micro-pores act as a nano reservoir in SnO_2_ to store absorbed water molecules; these water molecules can act as a proton-transporting channel across the membrane [62]. The bound water results in a copious amount of the hydroxyl group on its surface. The strong covalent Sn-O bond due to the high electronegativity difference between tin and oxygen leads to easy detachment of the surface hydroxyl groups and dissociates the protons to a greater extent and tends to dissociate the protons [63]. The same proton conduction mechanism is valid for ZrO_2_ and SiO_2_. However, the electronegativity difference between Si-O is lower than Sn-O but higher than Zr-O, resulting in the following trend for the conductivity values of the membranes: PVA-SiO_2_-Sn > PVA-SiO_2_-Si > PVA-SiO_2_-Zr, and the exactly reversed trend for the ASR values of the membranes, i.e., PVA-SiO_2_-Zr > PVA-SiO_2_-Si > PVA-SiO_2_-Sn. A similar trend for the conductivity and ASR was observed for vanadium-electrolyte-doped membranes. However, the conductivity values of 12.50, 11.42 and 15.00 mS cm^−1^ for vanadium-electrolyte-doped PVA-SiO_2_-Si, PVA-SiO_2_-Zr, and PVA-SiO_2_-Sn membranes were lower compared to acid-doped membranes, and area specific resistance values of 1.20, 1.27 and 1.00 ohm cm^2^ for vanadium-electrolyte-doped PVA-SiO_2_-Si, PVA-SiO_2_-Zr and PVA-SiO_2_-Sn were relatively higher than the acid-doped membranes. This can be blamed on the irreversible vanadium ions on the membrane matrix, which hinder the conduction of charge carrier ions. Additionally, the influence of the metal oxide layer on the surface charge of the membrane was confirmed by measuring the zeta potential in a neutral solution (1 mM KCl, ~7 pH). The recorded zeta potential values for PVA-SiO_2_-Si, PVA-SiO_2_-Zr and PVA-SiO_2_-Sn were −11.54, −7.70, and −14.93 mV (Figure S5). The values are in accordance with the conductivity data, suggesting the influence of the metal oxide coating on the membrane property.

The oxidative stability of the membranes was evaluated by determining the dimensional and weight change in a 2 M H_2_SO_4_ solution containing 1.5 M VO_2_^+^ ions. The dimensional variations, including length and width, as well as weight, were measured over a period of 168 h (7 days), and the data are presented in Figure S6a–c. The membranes displayed a high variation in weight and dimensions in the initial stages of the experiment but then remained relatively stable as the experiment concluded. The weights of PVA-SiO_2_-Si, PVA-SiO_2_-Zr and PVA-SiO_2_-Sn membranes initially increased by 28.0, 38.0 and 30.0% and gradually decreased to remain relatively constant with 24.0, 32.0 and 34.0% increases in weight for PVA-SiO_2_-Si, PVA-SiO_2_-Zr and PVA-SiO_2_-Sn on the final day. There was a mere ~10.0% increase in length for all the membranes initially, and thereafter, they remained constant till the conclusion of the experiment, while the change in width of all the membranes was observed over 4 days and, thereafter, remained constant. The change in dimension and weight for all the membranes can be attributed to the irreversible adsorption of vanadium ions and sorption of the electrolyte into the membrane matrix. More importantly, no membranes were found to be torn, and the mechanical strength of the membranes was found to be intact at the end of the oxidative stability experiments.

The effect of the metal oxide coating on vanadium permeation was studied with a two-compartment cell, as reported in our earlier studies [49,64]. The diffusion coefficient for V^3+^ and VO^2+^ cations for all the membranes was found to be lower than the state-of-art Nafion-117 in an identical experimental set-up. It is well known that hydrated oxides of highly charged metals are extremely stable in a corrosive environment, and at the same time, in acidic medium, these compounds display anion-exchange properties [65]. The membrane with the ZrO_2_ coating, i.e., PVA-SiO_2_-Zr, had the lowest permeability of 3.86 × 10^−8^ and 6.45 × 10^−8^ cm^2^ s^−1^ for V^3+^ and VO^2+^. It was one order fewer compared to Nafion-117. The diffusion of vanadium ions across PVA-SiO_2_-Sn and PVA-SiO_2_-Si was also slower than Nafion-117 (Table 1). The vanadium ion permeability for the PVA-SiO_2_-Si membrane was found to be 2.34 × 10^−7^ and 1.50 × 10^−7^ for V^3+^ and VO^2+^, respectively. The aqua complex formation of V^3+^ and VO^2+^ accelerates the diffusion of vanadium ions across the membranes [66]. Furthermore, the neutral complex of VOSO_4_ does not experience repulsion from the PVA-SiO_2_-Si membrane matrix and easily sneaks across the membranes [67], proving relatively high values for the vanadium ion diffusion coefficient. In the case of PVA-SiO_2_-Sn, the proton-storing ability of SnO_2_ prevents the sneaking of the neutral complex of VOSO_4_ across the membrane, resulting in a relatively low vanadium ion diffusion coefficient compared to PVA-SiO_2_-Si.

### VRFB Performance of Surface-Modified PVA-SiO_2_ Membranes

**Figure 5:**
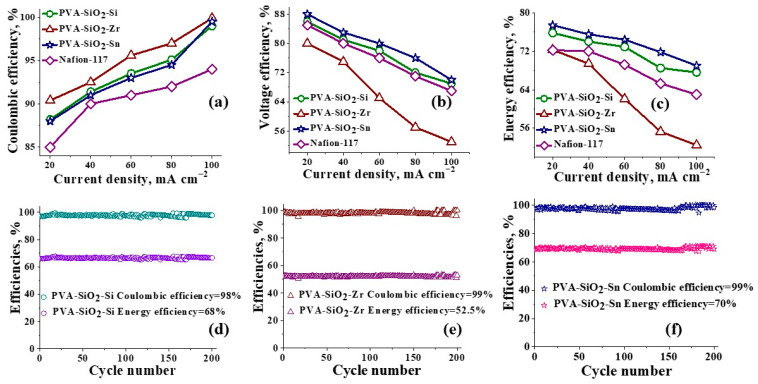
VRFB performance: rate capability and cycling test of the surface-modified PVA-SiO_2_ membranes, (**a**) Coulombic efficiencies, (**b**) voltage efficiencies and (**c**) energy efficiencies of PVA-SiO_2_-Si, PVA-SiO_2_-Zr, PVA-SiO_2_-Sn and Nafion-117. (**d**) PVA-SiO_2_-Si, (**e**) PVA-SiO_2_-Zr and (**f**) PVA-SiO_2_-Sn coulombic and energy efficiencies for continuous 200 charge/discharge cycles.

**Figure 6:**
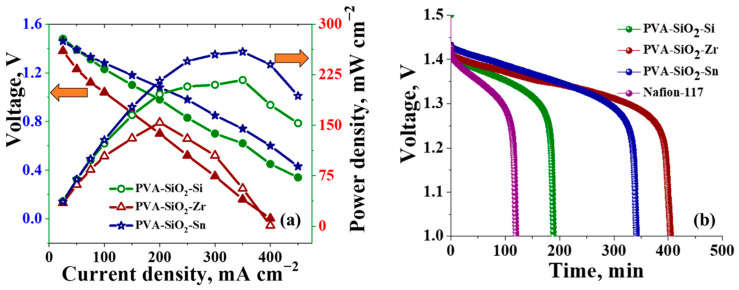
(**a**) Polarization curves and (**b**) self-discharge of VRFB assembled with PVA-SiO_2_-Si, PVA-SiO_2_-Zr and PVA-SiO_2_-Sn.

The rate capability, cycling test, open circuit potential and polarization curves for a single cell VRFB assembled with metal-oxide-coated membranes and its comparison with state-of-the-art Nafion-117 are presented in Figure 5 and Figure 6. Figure 5a depict the coulombic efficiencies of the membranes at different current densities (20–100 mA cm^2^); for all three membranes, there is an increase in CE with the increase in applied current density. This is caused due to the shortening of the charge/discharge time at higher current densities, which prevents the bulk leakage of vanadium ions during battery operation [68]. The VRFB with PVA-SiO_2_-Si recorded CE of 88.2, 91.4, 93.5, 95.1 and 99.0% at 20, 40, 60, 80 and 100 mA cm^−2^ current density. In identical testing conditions, PVA-SiO_2_-Zr recorded CE of 90.4, 92.5, 95.6, 97.0 and 99.9% at current densities of 20, 40, 60, 80 and 100 mA cm^−2^. Meanwhile, CE of 88.0, 91.0, 93.0, 94.5 and 99.5% was calculated for PVA-SiO_2_-Sn at current densities of 20, 40, 60, 80 and 100 mA cm^−2^. From the figure, it can be clearly seen that the CE of the synthesized membranes was better than Nafion-117 at every recorded current density. VRFB with Nafion-117 resulted in CE of 85.0, 90.0, 91.0, 92.0 and 94.0% at 20, 40, 60, 80 and 100 mA cm^−2^ current density, respectively.

Figure 5b represents the voltage efficiencies (VE) of the membranes. Unlike CE, VE is found to be decreasing with the increasing applied current density. Ohmic loss and high overpotential are blamed for this characteristic trend. Ohmic loss is defined as the product of internal resistance and applied current density [69]. As the voltage efficiency is highly related to conductivity of the membranes, the membrane with the highest conductivity PVA-SiO_2_-Sn displayed the highest voltage efficiencies of 88.0, 83.0, 80.0, 76.0 and 70.0% at current densities of 20, 40, 60, 80 and 100 mA cm^−2^. PVA-SiO_2_-Zr displayed comparatively poor VE of 80.0, 75.0, 65.0, 57.0 and 53.0% at 20, 40, 60, 80 and 100 mA cm^−2^ current density, as among the three prepared membranes, its conductivity was the least. For PVA-SiO_2_-Si, which had conductivity better than PVA-SiO_2_-Zr but less than PVA-SiO_2_-Sn, recorded VE of 86.0, 81.0, 78.0, 72.0 and 69.0% at 20, 40, 60, 80 and 100 mA cm^−2^ current density. Meanwhile, in the same testing environment, the calculated VE for Nafion-117 was found to be 85, 80, 76, 71 and 67.0% at 20, 40, 60, 80 and 100 mA cm^−2^ current density.

Energy efficiency (EE) is an important parameter used to evaluate the performance of the battery. It is calculated as the product of CE and VE. It can be seen from Figure 5c that the energy efficiencies of the membranes had a similar trend to that of their VE. The calculated EE for PVA-SiO_2_-Sn was 77.4, 75.4, 74.4, 71.8 and 69.0% at 20, 40, 60, 80 and 100 mA cm^−2^ current density. Whereas, for PVA-SiO_2_-Si, EE was found to be 75.8, 74.0, 72.9, 68.5 and 67.6% at 20, 40, 60, 80 and 100 mA cm^−2^ current density, and for PVA-SiO_2_-Zr, it was 72.3, 69.4, 62.1, 55.3 and 52.5% at 20, 40, 60, 80 and 100 mA cm^−2^ current density. Nafion-117 had 72.2, 72.0, 69.2, 65.3 and 63.0% at a current density of 20, 40, 60, 80 and 100 mA cm^−2^.

The calendar life of the membranes was determined with uninterrupted 200 charge/discharge cycles at 100 mA cm^−2^ (Figure 5d–f). The consistent efficiencies recorded for the membrane throughout the cycling test speak for the stability of the membranes for long-term application. The CE for all the prepared membranes was ~99.9%, which was ~5.3% higher than the CE of state-of-the-art Nafion 117 and 2.6% higher than the neat PVA-SiO_2_ membrane in identical testing environment (Figures S7 and S8). These results are in complete agreement with the lower vanadium permeability for metal-oxide-coated PVA-SiO_2_ membranes compared to the Nafion 117 membrane. The average voltage efficiency of ~70.0% for PVA-SiO_2_-Sn was ~4.5% higher than Nafion-117 and 5.3% higher than the neat non-coated PVA-SiO_2_ membrane, whereas the average voltage efficiency calculated for PVA-SiO_2_-Si and PVA-SiO_2_-Zr was found to be ~67.0 and ~53.0% over continuous 200 charge/discharge cycles, and the EE of PVA-SiO_2_-Sn, PVA-SiO_2_-Si and PVA-SiO_2_-Zr was ~70.0, ~67.0 and ~53.0%, respectively. Meanwhile, both Nafion-117 and neat PVA-SiO_2_ exhibited an EE of 63.0% (Figures S5 and S6). The highest EE of ~70.0% for the PVA-SiO_2_-Sn membrane was comparable to the recent literature. Polybenzimidazole-based polymers obtained via the incorporation of hydrophilic poly (ethylene glycol) methyl ether sidechains in a single-cell VRFB study exhibited an average of ~68.0% EE over long cycling at 140 mA cm^−2^ [70]. Zhai and group reported an average EE of ~75% over 100 cycles at 120 mA cm^−2^ for sulfonated poly (ether ether ketone) (SPEEK) [71]. The sulfonated polyimide molybdenum disulfide composite membrane delivered 66.9% EE at 80 mA cm^−2^ [72]. The sulfonated polyethylene-styrene cation exchange membrane recently reported by our group displayed an average EE of 63.0% energy at 140 mA cm^−2^ over 100 charge/discharge cycles [49]. A novel carboxyl-containing polyimide (PID) grafting sulfonated polyvinyl alcohol (SPVA) copolymer membrane, prepared by Xia and team [73], achieved an EE of 74.3 at 90 mA cm^−2^. It is worth mentioning that most of the membranes reported for VRFB contain strong cationic or anionic functional groups to achieve high ionic conduction and enhanced energy efficiencies. However, the membranes reported in this work are devoid of cationic/anionic functional groups; the high proton conduction of SnO_2_ on the membrane surface and well-defined ion conducting channels in the membrane matrix anchored by silica resulted in a high EE. Whereas, in the case of PVA-SiO_2_-Si, there is the possibility of the extension of interconnected proton-conducting channels in the membrane matrix with the silica on the membrane surface, which may have eased the conductivity of the charge carriers through the membrane to deliver an acceptable EE at a high current density. ZrO_2_ on the membrane surface of PVA-SiO_2_-Zr created an unavoidable resistance to charge balancing ions and, hence, showed poor EE. However, the excellent vanadium impermeability of the ZrO_2_ layer resulted in only 0.58% average capacity decay per cycle for PVA-SiO_2_-Zr as compared to the average capacity decay per cycle of 0.74, 0.85 and 0.94% for PVA-SiO_2_-Sn, PVA-SiO_2_-Si and Nafion-117.

Polarization curves for the VRFB assembled with PVA-SiO_2_-Si, PVA-SiO_2_-Zr and PVA-SiO_2_-Sn are depicted in Figure 6a. The initial voltage drop with the increase in applied current density for PVA-SiO_2_-Zr was governed by activation losses at initial current densities; voltage drop at the linear part of the curve for all the membranes is caused by the ohmic resistance; and finally, the mass transfer losses at high current densities mark the complete exhaustion of redox active species at the electrode surface [74]. However, the combined losses can be minimized by engineering the electrode surfaces, as discussed elsewhere [75,76]. The VRFB with a PVA-SiO_2_-Sn membrane exhibited the highest peak power density of 260 mW cm^−2^, reflecting its high proton conduction. The peak power density of PVA-SiO_2_-Si was 215 mW cm^−2^, suggesting a good proton conduction property of the membrane even at higher current densities. However, the PVA-SiO_2_-Zr membrane displayed a poor power density of a mere 154 mW cm^−2^, which can be attributed to the high resistance of the ZrO_2_ layer on the membrane surface. Nevertheless, the ZrO_2_ layer created a torturous path for the diffusion of vanadium ions across the membrane, which can be interpreted from its high self-discharge time of 405 min. Meanwhile, the self-discharge curves recorded for PVA-SiO_2_-Sn and PVA-SiO_2_-Si were 342 and 190 min, respectively, were in line with vanadium ion permeability statistics. Post-performance analysis of the membranes was evaluated by determining through-plane conductivity and sulfuric acid (2 M) content. The through-plane conductivity of the membranes, PVA-SiO_2_-Si, PVA-SiO_2_-Zr and PVA-SiO_2_-Sn after battery performance was found to be 12.90, 9.95, and 18.94 mS cm^−1^, and its corresponding sulfuric acid (2 M) uptake was 87, 80 and 87%. The data reflect (Table S1) a more than 80% retention of conductivity and 90% retention of sulfuric acid (2 M) uptake for the membranes after detailed battery performance in the oxidative environment. This suggests the potential of the methodology in membrane modification and its best utility in VRFB application.

In summary, a metal oxide coating on a thermally crosslinked PVA-SiO_2_ membrane surface turned out to be an effective strategy to develop high performance separators. The high proton conduction and low area specific resistance of PVA-SiO_2_-Sn and PVA-SiO_2_-Si membranes can serve as a potential membrane for proton exchange membrane fuel cell (PEMFC) and redox flow battery (RFB) applications while the excellent ion barricading property of PVA-SiO_2_-Zr can play a pivotal role in redox flow battery (RFB), acid recovery from industrial effluent with dissolved metal salts and ion-selective electrodialysis.

## 4. Conclusions

To summarize, a thermally crosslinked PVA-SiO_2_ composite was synthesized and the surface of the membrane was modified with a skin layer of metal oxide. Coatings of SiO_2_, ZrO_2_ and SnO_2_ via an acid-catalyzed solution-gelation technique resulted in three membranes, namely, PVA-SiO2-Si, PVA-SiO_2_-Zr and PVA-SiO_2_-Sn. The membranes displayed high content due to the hydrophilic PVA-SiO_2_ matrix and a hygroscopic metal oxide surface layer. Nonetheless, the membranes were found to be dimensionally stable with acceptable mechanical strength and ionic conductivity. Fascinatingly, the membranes showcased excellent oxidative stability in a 2M H_2_SO_4_ solution containing 1.5 M VO_2_^+^ ions. We believe that the effective thermal crosslinking of the membranes and metal oxide coating restricted the permeation of hydrogen peroxide into the membrane matrix. The vanadium ion permeability study in a charge-balanced two-compartment cell suggests low diffusion of vanadium ions across the membranes as compared to Nafion-117 in identical environmental set-up. In the single-cell VRFB experiments, the prepared membranes displayed higher CE than Nafion 117 in the rate capability testing. The stable efficiencies at 100 mA cm^−2^ over 200 charge/discharge cycles for the membranes speak to the efficacy of the membrane for a long service life. The VRFB assembled with PVA-SiO_2_-Sn membrane delivered CE of 99.9% and EE of 70.0%, as compared to 94.6% CE and 63.0% EE for Nafion 117 in identical cell-testing conditions. In the polarization curve experiments, PVA-SiO_2_-Sn, PVA-SiO_2_-Si and PVA-SiO_2_-Zr had the highest peak power density of 260, 215 and 154 mW cm^−2^. The battery performance of the membranes suggests facile surface modification and synthetic procedures could afford an efficient polyelectrolyte membrane material for VRFB application. Furthermore, with the proper choice of design and materials, advanced ion exchange membranes can be tuned for higher electrochemical performance in energy device and separation/purification technology.

## Figures and Tables

**Table 1:** Vanadium ion diffusion coefficient across surface modified PVA-SiO_2_ membranes and Nafion-117.

Membranes	V^3+^ Diffusion Coefficient (cm^2^ s^−1^)	VO^2+^ Diffusion Coefficient (cm^2^ s^−1^)
PVA-SiO_2_-Si	2.34 × 10^−7^	1.50 × 10^−7^
PVA-SiO_2_-Zr	3.86 × 10^−8^	6.45 × 10^−8^
PVA-SiO_2_-Sn	1.47 × 10^−7^	1.09 × 10^−7^
Nafion-117	8.60 × 10^−7^	1.04 × 10^−6^

## Data Availability

Data is contained within the article or Supplementary Material.

## Supplementary Materials

The following supporting information can be downloaded at: https://www.mdpi.com/article/10.3390/membranes13060574/s1, Figure S1: a–c, Cross-sectional SEM images of the membranes. PVA-SiO_2_-Si, b. PVA-SiO_2_-Zr and c. PVA-SiO_2_-Sn; Figure S2: a. SEM image of PVA-SiO2-Si and b, c and d. corresponding elemental mapping of Silica, oxygen, and carbon of PVA-SiO_2_-Si; Figure S3: a. SEM image of PVA-SiO_2_-Zr and b, c, d and e. corresponding elemental mapping of Silica, oxygen, carbon, and zirconium of PVA-SiO_2_-Zr; Figure S4: a. SEM image of PVA-SiO_2_-Sn and b, c, d and e. corresponding elemental mapping of Silica, oxygen, carbon, and tin of PVA-SiO_2_-Sn; Figure S5: Zeta potential values of metal oxide coated PVA-SiO_2_ membranes; Figure S6: a. Weight; b. length and c. width of PVA-SiO_2_-Si, PVA-SiO_2_-Zr and PVA-SiO_2_-Sn membranes as function of immersing time in 1.5 M VO^2+^ dissolved in 2 M H_2_SO_4_ solutions; Figure S7: Cycling test of Nafion-117 at 100 mA cm^−2^; Figure S8: Cycling test of neat PVA-SiO_2_ membrane at 100 mA cm^−2^; Table S1: Comparison of through-plane conductivity and sulfuric acid (2 M) uptake values of the metal oxide coated membranes before and after VRFB study.

## References

[1] Nagarale R.K., Gohil G.S., Shahi V.K. (2006). Recent developments on ion-exchange membranes and electro-membrane processes. Adv. Colloid Interface Sci..

[2] Ran J., Wu L., He Y., Yang Z., Wang Y., Jiang C., Ge L., Bakangura E., Xu T. (2017). Ion exchange membranes: New developments and applications. J. Membr. Sci..

[3] Varcoe J.R., Atanassov P., Dekel D.R., Herring A.M., Hickner M.A., Kohl P.A., Kucernak A.R., Mustain W.E., Nijmeijer K., Scott K. (2014). Anion-exchange membranes in electrochemical energy systems. Energy Environ. Sci..

[4] Hickner M.A., Herring A.M., Coughlin E.B. (2013). Anion Exchange Membranes: Current Status and Moving Forward. J. Polym. Sci. B Polym. Phys..

[5] Stenina I.A., Yaroslavtsev A.B. (2021). Ionic Mobility in Ion-Exchange Membranes. Membranes.

[6] Luo T., Abdu S., Wessling M. (2018). Selectivity of ion exchange membranes: A review. J. Membr. Sci..

[7] Li X., Zhang H., Mai Z., Zhang H., Vankelecom I. (2011). Ion exchange membranes for vanadium redox flow battery (VRB) applications. Energy Environ. Sci..

[8] Strathmann H., Grabowski A., Eigenberger G. (2013). Ion-Exchange Membranes in the Chemical Process Industry. Ind. Eng. Chem. Res..

[9] Remiš T., Bělský P., Andersen S.M., Tomáš M., Kadlec J., Kovářík T. (2020). Preparation and Characterization of Poly(Vinyl Alcohol) (PVA)/SiO_2_, PVA/Sulfosuccinic Acid (SSA) and PVA/SiO_2_/SSA Membranes: A Comparative Study. J. Macromol. Sci. B.

[10] Kariduraganavar M.Y., Nagarale R.K., Kittur A.A., Kulkarni S.S. (2006). Ion-exchange membranes: Preparative methods for electrodialysis and fuel cell applications. Desalination.

[11] Peighambardoust S.J., Rowshanzamir S., Amjadi M. (2010). Review of the proton exchange membranes for fuel cell applications. Int. J. Hydrogen Energy.

[12] Weng G., Ouyang K., Lin X., Xue J., Wang H. (2021). Proton conducting membranes for hydrogen and ammonia production. React. Chem. Eng..

[13] Bai H., Ho W.S.W. (2011). Recent developments in fuel-processing and proton-exchange membranes for fuel cells. Polym. Int..

[14] Wycisk R., Pintauro P.N., Park J.W. (2014). New developments in proton conducting membranes for fuel cells. Curr. Opin. Chem. Eng..

[15] Jannasch P. (2003). Recent developments in high-temperature proton conducting polymer electrolyte membranes. Curr. Opin. Colloid Interface Sci..

[16] Jiang S., Sun H., Wang H., Ladewig B.P., Yao Z. (2021). A comprehensive review on the synthesis and applications of ion exchange membranes. Chemosphere.

[17] Xu T. (2005). Ion exchange membranes: State of their development and perspective. J. Membr. Sci..

[18] Pawar C.M., Sreenath S., Bhatt B., Nikumbe D.Y., Saleha W.F.G., Nagarale R.K. (2023). Surface modification, counter-ion exchange effect on thermally annealed sulfonated poly (ether ether ketone) membranes for vanadium redox flow battery. Colloids Surf. A Physicochem. Eng. Asp..

[19] Kobuchi Y., Motomura H., Noma Y., Hanada F. (1986). Application of ion exchange membranes to the recovery of acids by diffusion dialysis. J. Membr. Sci..

[20] Yaroslavtsev A.B. (2013). Perfluorinated Ion-Exchange Membranes. Polym. Sci. Ser. A.

[21] Kusoglu A., Weber A.Z. (2017). New Insights into Perfluorinated Sulfonic-Acid Ionomers. Chem. Rev..

[22] Smitha B., Sridhar S., Khan A.A. (2005). Solid polymer electrolyte membranes for fuel cell applications-a review. J. Membr. Sci..

[23] Yee R.S.L., Rozendal R.A., Zhang K., Ladewig B.P. (2012). Cost effective cation exchange membranes: A review. Chem. Eng. Res. Des..

[24] Hickner M.A., Ghassemi H., Kim Y.S., Einsla B.R., McGrath J.E. (2004). Alternative Polymer Systems for Proton Exchange Membranes (PEMs). Chem. Rev..

[25] Kang M.-S., Choi Y.-J., Choi I.-J., Yoon T.-H., Moon S.-H. (2003). Electrochemical characterization of sulfonated poly(arylene ether sulfone) (S-PES) cation-exchange membranes. J. Membr. Sci..

[26] Pagels M.K., Adhikari S., Walgama R.C., Singh A., Han J., Shin D., Bae C. (2020). One-Pot Synthesis of Proton Exchange Membranes from Anion Exchange Membrane Precursors. ACS Macro Lett..

[27] Prajapati P.K., Reddy N.N., Nimiwal R., Singh P.S., Adimurthy S., Nagarale R.K. (2020). Polyaniline@porous polypropylene for efficient separation of acid by diffusion dialysis. Sep. Purif. Technol..

[28] Aparicio G.M., Vargas R.A., Bueno P.R. (2019). Protonic conductivity and thermal properties of cross-linked PVA/TiO_2_ nanocomposite polymer membranes. J. Non Cryst. Solids.

[29] Jin Y., Diniz da Costa J.C., Lu G.Q. (2007). Proton conductive composite membrane of phosphisilicate and polyvinyl alcohol. Solid State Ion..

[30] Nagarale R.K., Shahi V.K., Rangarajan R. (2005). Preparation of polyvinyl alcohol–silica hybrid heterogeneous anion-exchange membranes by sol–gel method and their characterization. J. Membr. Sci..

[31] Binsu V.V., Nagarale R.K., Shahi V.K. (2005). Phosphonic acid functionalized aminopropyl triethoxysilane-PVA composite material: Organic–inorganic hybrid proton-exchange membranes in aqueous media. J. Mater. Chem..

[32] Kamjornsupamitr T., Sangthumchai T., Youngme S., Martwiset S. (2018). Proton conducting composite membranes from crosslinked poly(vinyl alcohol) and poly(styrene sulfonic acid)-functionalized silica nanoparticles. Int. J. Hydrogen Energy.

[33] Sgreccia E., Narducci R., Knauth P., Luisa Di Vona M. (2021). Silica Containing Composite Anion Exchange Membranes by Sol–Gel Synthesis: A Short Review. Polymers.

[34] Zhang Y., Guo M., Yan H., Pan G., Xu J., Shi Y., Liu Y. (2014). Novel organic–inorganic hybrid composite membranes for nanofiltration of acid and alkaline media. RSC Adv..

[35] Wang Y., Wang D., Wang J., Wang L. (2020). Preparation and characterization of a sol-gel derived silica/PVA-Py hybrid anion exchange membranes for alkaline fuel cell application. J. Electroanal. Chem..

[36] Dodda J.M., Bělský P., Chmelar J., Remis T., Smolna K., Tomáš M., Kullova L., Kadlec J. (2015). Comparative study of PVA/SiO_2_ and PVA/SiO_2_/glutaraldehyde (GA) nanocomposite membranes prepared by single-step solution casting method. J. Mater. Sci..

[37] Nagarale R.K., Gohil G.S., Shahi V.K., Rangarajan R. (2004). Organic-Inorganic Hybrid Membrane: Thermally Stable Cation-Exchange Membrane Prepared by the Sol-Gel Method. Macromolecules.

[38] Panero S., Fiorenza P., Navarra M.A., Romanowska J., Scrosati B. (2005). Silica-Added, Composite Poly(vinyl alcohol) Membranes for Fuel Cell Application. J. Electrochem. Soc..

[39] Beydaghi H., Javanbakht M., Badiei A. (2014). Cross-linked poly(vinyl alcohol)/sulfonated nanoporous silica hybrid membranes for proton exchange membrane fuel cell. J. Nanostruct. Chem..

[40] Nagarale R.K., Shin W., Singh P.K. (2010). Progress in ionic organic-inorganic composite membranes for fuel cell applications. Polym. Chem..

[41] Hegde S.N., Manuvalli B.B., Kariduraganavar M.Y. (2022). A Unique Approach for the Development of Hybrid Membranes by Incorporating Functionalized Nanosilica into Crosslinked sPVA/TEOS for Fuel Cell Applications. ACS Appl. Energy Mater..

[42] Panawong C., Tasari S., Saejueng P., Budsombat S. (2022). Composite proton conducting membranes from crosslinked poly(vinyl alcohol)/chitosan and silica particles containing poly(2-acrylamido-2-methyl-1-propansulfonic acid). J. Appl. Polym. Sci..

[43] Beydaghi H., Javanbakht M., Amoli H.S., Badiei A., Khaniani Y., Ganjali M.R., Norouzi P., Abdouss M. (2011). Synthesis and characterization of new proton conducting hybrid membranes for PEM fuel cells based on poly(vinyl alcohol) and nanoporous silica containing phenyl sulfonic acid. Int. J. Hydrogen Energy.

[44] Murmu R., Roy D., Patra S.C., Sutar H., Senapati P. (2022). Preparation and characterization of the SPEEK/PVA/Silica hybrid membrane for direct methanol fuel cell (DMFC). Polym. Bull..

[45] Nagarale R.K., Gohil G.S., Shahi V.K., Rangarajan R. (2005). Preparation of organic–inorganic composite anion-exchange membranes via aqueous dispersion polymerization and their characterization. J. Colloid Interface Sci..

[46] Ying Y.P., Kamarudin S.K., Masdar M.S. (2018). Silica-related membranes in fuel cell applications: An overview. Int. J. Hydrogen Energy.

[47] Guo R., Ma X., Hu C., Jiang Z. (2007). Novel PVA-silica nanocomposite membrane for pervaporative dehydration of ethylene glycol aqueous solution. Polymer.

[48] Sreenath S., Sharma N.K., Nagarale R.K. (2020). Alkaline all iron redox flow battery with a polyethylene/poly(styrene-co-divinylbenzene) interpolymer cation-exchange membrane. RSC Adv..

[49] Sreenath S., Pawar C.M., Bavdane P., Nikumbe D.Y., Nagarale R.K. (2022). A sulfonated polyethylene–styrene cation exchange membrane: A potential separator material in vanadium redox flow battery applications. Energy Adv..

[50] Cao L., Kronander A., Tang A., Wang D.-W., Skyllas-Kazacos M. (2016). Membrane Permeability Rates of Vanadium Ions and Their Effects on Temperature Variation in Vanadium Redox Batteries. Energies.

[51] Binsu V.V., Nagarale R.K., Shahi V.K., Ghosh P.K. (2006). Studies on N-methylene phosphonic chitosan/poly(vinyl alcohol) composite proton-exchange membrane. React. Funct. Polym..

[52] Nasef M.M., Fujigaya T., Abouzari-Lotf E., Nakashima N., Yang Z. (2016). Enhancement of performance of pyridine modified polybenzimidazole fuel cell membranes using zirconium oxide nanoclusters and optimized phosphoric acid doping level. Int. J. Hydrogen Energy.

[53] Vianna de Aguiar L.C., Gomes de Ramos Filho F., Dahmouche K., Perez G., Archanjo B.S., Kawaguti C.A., Gomes A.D.-S. (2020). SPEEK-Tin dioxide proton conducting membranes: Effect of modifying agent of tin dioxide particles surface. J. Appl. Polym. Sci..

[54] Peng Z., Kong L.X. (2007). A thermal degradation mechanism of polyvinyl alcohol/silica nanocomposites. Polym. Degrad. Stab..

[55] Kima D.S., Park H.B., Rhim J.W., Lee Y.M. (2004). Preparation and characterization of crosslinked PVA/SiO_2_ hybrid membranes containing sulfonic acid groups for direct methanol fuel cell applications. J. Membr. Sci..

[56] Rawat R.K., Chauhan P. (2021). A novel approach to achieve the tin (II) oxide based proton conductive sensor for ammonia detection at room temperature. Mater. Lett..

[57] Hara S., Takano S., Miyayama M. (2004). Proton-Conducting Properties and Microstructure of Hydrated Tin Dioxide and Hydrated Zirconia. J. Phys. Chem. B.

[58] Hara S., Sakamoto H., Miyayama M., Kudo T. (2002). Proton-conducting properties of hydrated tin dioxide as an electrolyte for fuel cells at intermediate temperature. Solid State Ion..

[59] Marrero J.C., Gomes A.D.-S., Hui W.S., Filho J.C.D., Silva de Oliveira V. (2017). Sulfonation degree effect on ion-conducting SPEEK-titanium oxide membranes properties. Polímeros.

[60] D’Epifanio A., Mecheri B., Passarini G., Traversa E., Miyayama M., Licoccia S. (2006). Proton Conducting Composite Membranes from Polyether Ether Ketone and Hydrated Metal Oxides. ECS Trans..

[61] Mecheri B., D’Epifanio A., Pisani L., Chen F., Traversa E., Weise F.C., Greenbaum S., Licoccia S. (2009). Effect of a Proton Conducting Filler on the Physico-Chemical Properties of SPEEK Based Membranes. Fuel Cells.

[62] Chen F., Mecheri B., D’Epifanio A., Traversa E., Licoccia S. (2010). Development of Nafion/TinOxide Composite MEA for DMFC Applications. Fuel Cells.

[63] Kumar G.G., Shin J., Nho Y.-C., Hwang I.S., Fei G., Kim A.R., Nahm K.S. (2010). Irradiated PVdF-HFP–tin oxide composite membranes for the applications of direct methanol fuel cells. J. Membr. Sci..

[64] Sreenath S., Nayanthara P.S., Pawar C.M., Nikumbe D.Y., Bhatt B., Chaudhari J.C., Nagarale R.K. (2022). High-Capacity Retention Thermally Reinforced Pore-Filled Anion Exchange Membrane for All-Vanadium Flow Batteries. ACS Appl. Energy Mater..

[65] Dzyaz’ko Y.S., Belyakov V.N., Stefanyak N.V., Vasilyuk S.L. (2006). Anion-Exchange Properties of Composite Ceramic Membranes Containing Hydrated Zirconium Dioxide. Russ. J. Appl. Chem..

[66] Vijayakumar M., Bhuvaneswari M.S., Nachimuthu P., Schwenzer B., Kim S., Yang Z., Liu J., Graff G.L., Thevuthasan S., Hu J. (2011). Spectroscopic investigations of the fouling process on Nafion membranes in vanadium redox flow batteries. J. Membr. Sci..

[67] Strehlow H., Wendt H. (1963). Fast ionic reactions in solution. IV. The formation of the vanadyl sulfate complex in aqueous solution. Inorg. Chem..

[68] Sreenath S., Nayanthara P.S., Pawar C.M., Noufal M.C., Nagarale R.K. (2021). Phenolic triamine dangling poly(VDF-co-HFP) anion exchange membrane for all aqueous organic redox flow battery. J. Energy Storage.

[69] Bavdane P.P., Sreenath S., Nikumbe D.Y., Pawar C.M., Kuzhiyil M.C., Nagarale R.K. (2022). N-Sulfonated Poly(arylene-oxindole) for Vanadium Redox Flow Battery Applications. ACS Appl. Energy Mater..

[70] Chen Y., Li A., Xiong P., Xiao S., Sheng Z., Peng S., He Q. (2022). Three birds with one stone: Microphase separation induced by densely grafted short chains in ion conducting membranes. J. Membr. Sci..

[71] Zhai S., Jia X., Lu Z., Ai Y., Liu X., Lin J., He S., Wang Q., Chen L. (2022). Highly ion selective composite proton exchange membranes for vanadium redox flow batteries by the incorporation of UiO-66-NH2 threaded with ion conducting polymers. J. Membr. Sci..

[72] Li J., Zhang Y., Zhang S., Huang X. (2015). Sulfonated polyimide/s-MoS2 composite membrane with high proton selectivity and good stability for vanadium redox flow battery. J. Membr. Sci..

[73] Xia Y., Liu B., Wang Y. (2019). Effects of covalent bond interactions on properties of polyimide grafting sulfonated polyvinyl alcohol proton exchange membrane for vanadium redox flow battery applications. J. Power Sources.

[74] Sharma J., Khan H., Upadhyay P., Kothandaraman R., Kulshrestha V. (2023). Stable Poly(2,6-dimethyl-1,4-phenylene ether) Based Cross-Linked Cationic Polyelectrolyte Membrane with Ionic Microstructure Modification for Efficient VRFB Performance. ACS Appl. Energy Mater..

[75] Wei L., Zhao T.S., Zhao G., An L., Zeng L. (2016). A high-performance carbon nanoparticle-decorated graphite felt electrode for vanadium redox flow batteries. Appl. Energy.

[76] Wei L., Xiong C., Jiang H.R., Fan X.Z., Zhao T.S. (2020). Highly catalytic hollow Ti_3_C_2_T_x_ MXene spheres decorated graphite felt electrode for vanadium redox flow batteries. Energy Storage Mater..

