# Supplementary material for: Proton Conducting Organic-Inorganic Composite Membranes for All-Vanadium Redox Flow Battery
Source: Membranes (Basel). 2023 Jun 1;13(6):574. doi: 10.3390/membranes13060574 (PMC10303046; doi:10.3390/membranes13060574)
Supplement: Supplementary file 1 [file membranes-13-00574-s001.zip › membranes-2391299-supplementary.pdf]

Supplementary Material

Proton Conducting Organic-Inorganic Composite Membranes for All-Vanadium Redox Flow Battery

Sooraj Sreenath [1,2], Nayanthara P. Sreelatha [1], Chetan M. Pawar [1,2], Vidhiben Dave [1,2], Bhavana Bhatt [1], Nitin G. Borle [1] and Rajaram Krishna Nagarale [1,2,*]

[1] Electro Membrane Processes Laboratory, Membrane Science and Separation Technology Division, CSIR-Central Salt and Marine Chemicals Research Institute, Bhavnagar 364002, India; sooraj4993@gmail.com (S.S.); nayantharabiju2015@gmail.com (N.P.S.); chetanpawar085@gmail.com (C.M.P.); vidhidave009@gmail.com (V.D.); bhavanabhatt26@gmail.com (B.B.); nitinborle34@gmail.com (N.G.B.)

[2] Academy of Scientific and Innovative Research (AcSIR), Ghaziabad 201002, India

* Correspondence: rknagarale@csmcri.res.in

Supplementary Figures and Table

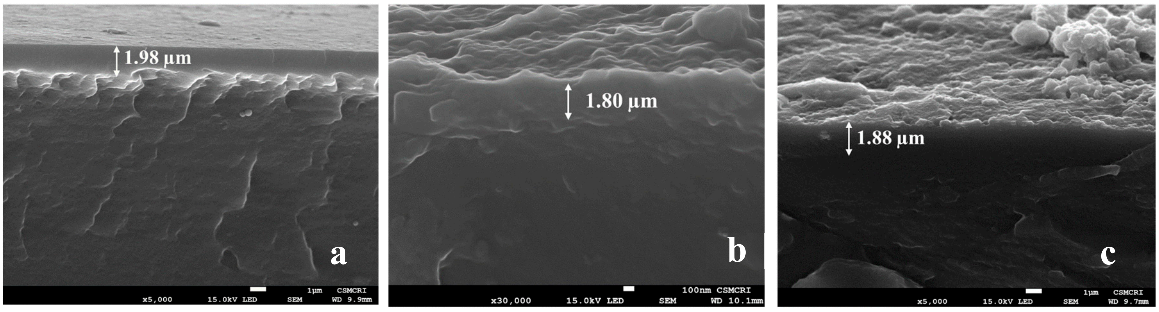

Figure S1. a-c, Cross-sectional SEM images of the membranes. PVA-SiO_2-Si, b. PVA-SiO_2-Zr and c. PVA-SiO_2-Sn.

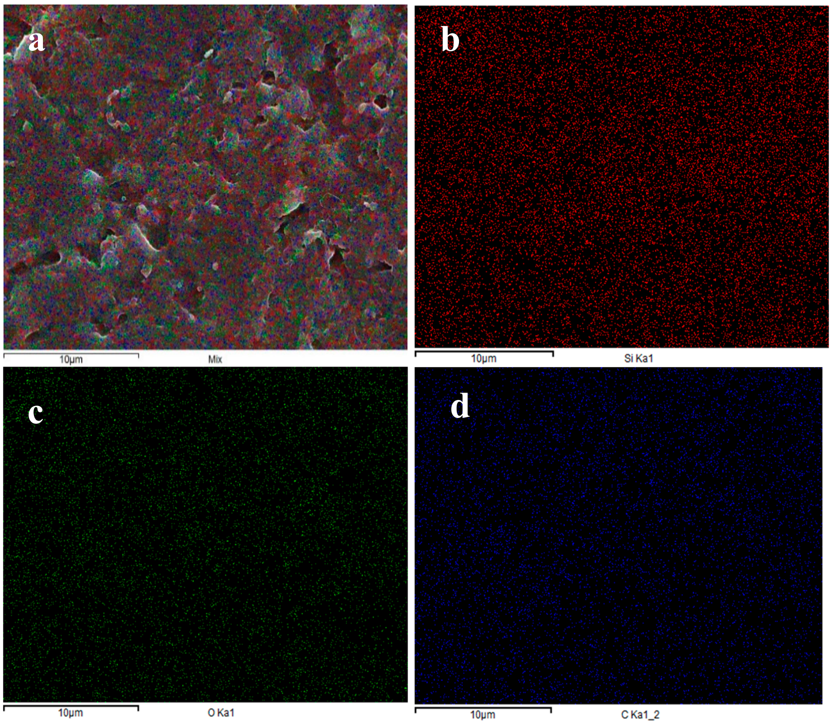

Figure S2: a. SEM image of PVA-SiO_2-Si and b–d. corresponding elemental mapping of Silica, oxygen, and carbon of PVA-SiO_2-Si.

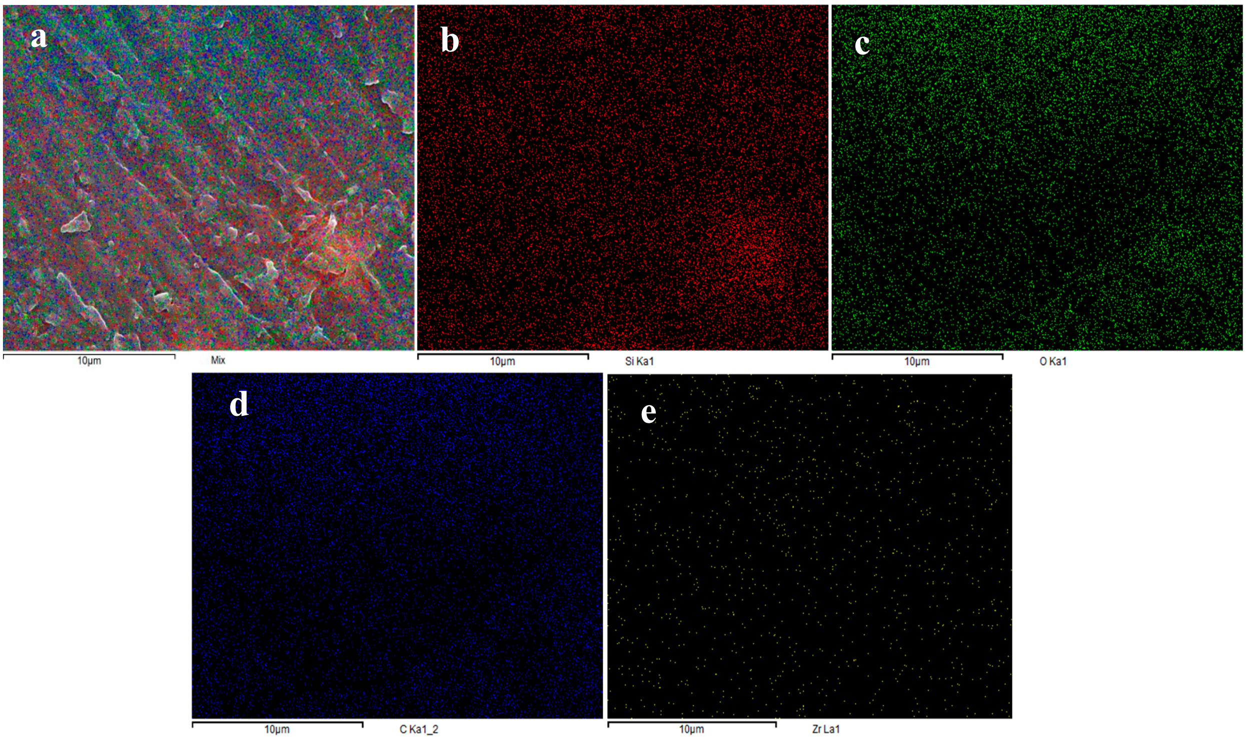

Figure S3: a. SEM image of PVA-SiO_2-Zr and b–e. corresponding elemental mapping of Silica, oxygen, carbon, and zirconium of PVA-SiO_2-Zr.

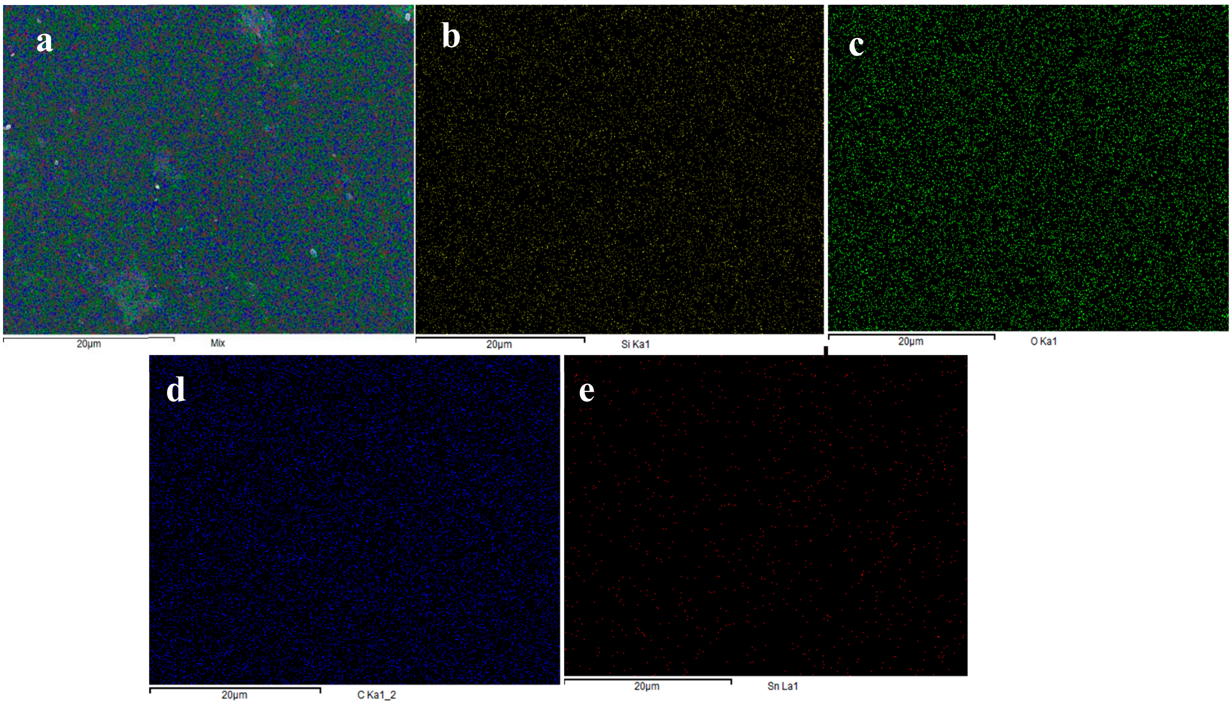

Figure S4: a. SEM image of PVA-SiO_2-Sn and b–e. corresponding elemental mapping of Silica, oxygen, carbon, and tin of PVA-SiO_2-Sn.

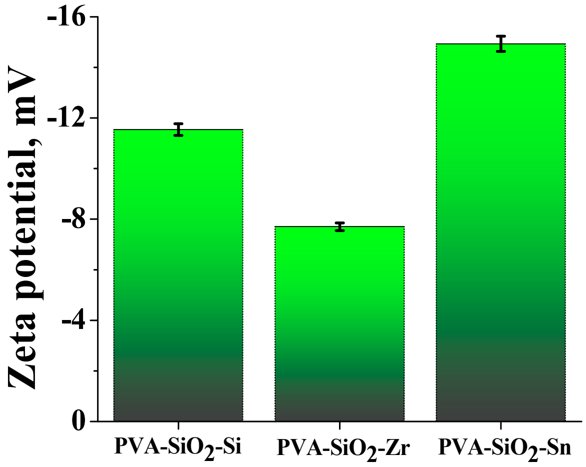

Figure S5. Zeta potential values of metal oxide coated PVA-SiO_2 membranes

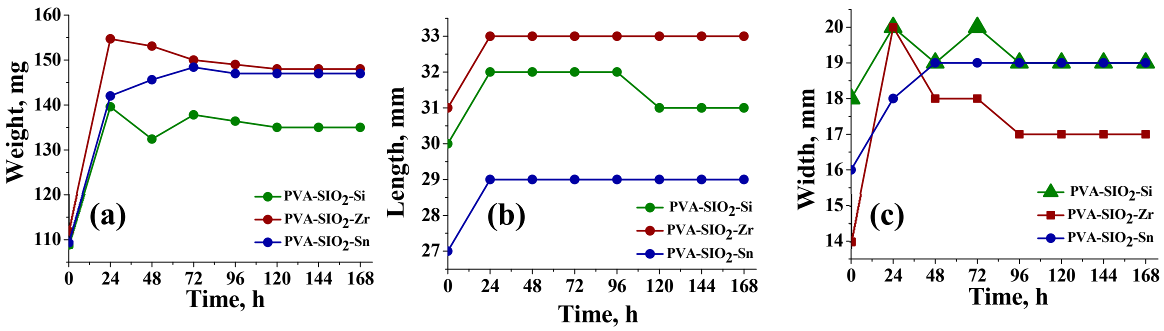

Figure S6: a. Weight; b. length and c. width of PVA-SiO2-Si, PVA-SiO2-Zr and PVA-SiO2-Sn membranes as function of immersing time in 1.5 M VO_2^+ dissolved in 2 M H_2SO_4 solutions.

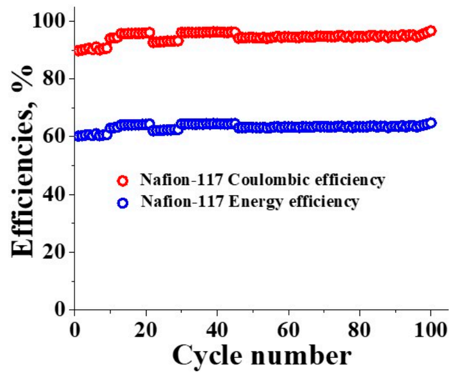

Figure S7. Cycling test of Nafion-117 at 100 mA cm^{-2}

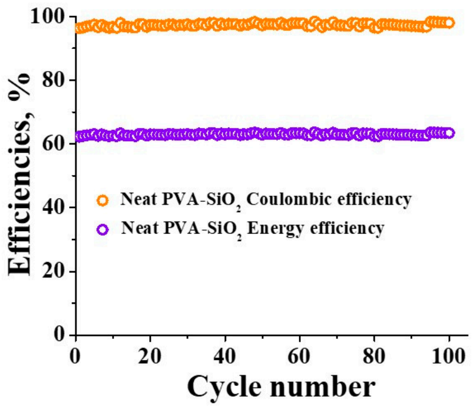

Figure S8. Cycling test of neat PVA-SiO2 membrane at 100 mA cm^{-2}

Table S1: Comparison of through-plane conductivity and sulfuric acid (2 M) uptake values of the metal oxide coated membranes before and after VRFB study.

Membranes code	Conductivity (mS cm^{-1})		Conductivity retention after VRFB (%)	2 M sulfuric acid uptake (%)		Water uptake retention after VRFB (%)
	Before VRFB	After VRFB		Before VRFB	After VRFB	
PVA-SiO_2-Si	15.00	12.90	86.00	95.0	87.0	91.57
PVA-SiO_2-Zr	12.28	9.95	81.02	86.0	80.0	93.02
PVA-SiO_2-Sn	20.27	18.94	93.43	89.0	87.0	97.75
